# Removal of Breast during Hypnotic Sleep

**Published:** 1890-11

**Authors:** 


### Removal of Breast During Hypnotic Sleep.

Dr. Schmeltz, of Nice, has recently (*Gazette Medicale de Strasbourgh*, July 1), recorded a case in which he removed a sarcomatous breast during anæsthesia caused by hypnotism. The patient was a girl, aged 20, who was easily thrown into the hypnotic sate. The operation was performed in the presence of Drs. Lauza and Barriera, and the entire organ, together with the aponeurosis of the pectoralis major was removed by the oval incision. Five drainage tubes were inserted, and the wound was closed with thirty-two metallic sutures. The operation lasted an hour. The patient remained absolutely insensible ; in a condition of the deepest anæsthesia, such as is only seen after large doses of chloroform. Dr. Schmeltz says : "I operated very slowly and quite at my ease ; the patient even tried to encourage me by her words ; she seemed very gay, and laughed loudly from time to time as if to show me that she felt no pain. In order to make the operation easier for me she turned herself about so as to place herself in the most favorable position, keeping her right arm stretched out so that no assistance was required to keep it steady." She was kept under observation the rest of the day, and having been told not to feel pain and to have a good night, she obeyed these instructions in the most docile manner. The wound was completely healed on the fifteenth day. The only symptom worth mentioning, which Dr. Schmeltz observed in the patient during the operation, was great pallor of the countenance, without any dilatation of the pupil or weakening of the pulse. The tumor weighed two kilograms.—*Brit. Med. Journal.*

